# Application of Hybrid Functional Groups to Predict ATP Binding Proteins

**DOI:** 10.1155/2014/581245

**Published:** 2014-01-08

**Authors:** Andreas N. Mbah

**Affiliations:** Center for Bioinformatics & Computational Biology, Department of Biology, Jackson State University, Jackson, MS 39217, USA

## Abstract

The ATP binding proteins exist as a hybrid of proteins with Walker A motif and universal stress proteins (USPs) having an alternative motif for binding ATP. There is an urgent need to find a reliable and comprehensive hybrid predictor for ATP binding proteins using whole sequence information. In this paper the open source LIBSVM toolbox was used to build a classifier at 10-fold cross-validation. The best hybrid model was the combination of amino acid and dipeptide composition with an accuracy of 84.57% and Mathews correlation coefficient (MCC) value of 0.693. This classifier proves to be better than many classical ATP binding protein predictors. The general trend observed is that combinations of descriptors performed better and improved the overall performances of individual descriptors, particularly when combined with amino acid composition. The work developed a comprehensive model for predicting ATP binding proteins irrespective of their functional motifs. This model provides a high probability of success for molecular biologists in predicting and selecting diverse groups of ATP binding proteins irrespective of their functional motifs.

## 1. Introduction

Recent advances in the next generation sequencing and human genome projects have resulted in rapid increase of protein sequences, thus widening the protein sequence-structure gap [1, 2], leading to diverse protein functions from common family. Computation prediction tools for predicting protein structure and function are highly needed to narrow the widening gap [3]. The ATP binding proteins (ATP-BPs) are a diverse family of proteins in terms of amino acid sequences, function, and their three-dimensional structures. These proteins hydrolyze ATP to provide the energy necessary to drive biochemical reactions in the cell [4]. There are two distinct functional groups of ATP binding proteins.

The first functional group has the Walker A motif [GXXXXGK (T/S) or G-4X-GK (T/S)] in their sequences for ATP binding [5]. Many members are transmembrane proteins and are responsible for transporting a wide variety of substrates across extra- and intracellular membranes [6]. The biochemical functions of ATP binding proteins are well exhibited within the ABC transporters group. In bacteria cell, ABC transporters pump substances such as sugars, vitamins, and metal ions into the cell, while in eukaryotes they transport molecules out of the cell [7]. They are also known to transport lipids and play a protective role to the developing fetus against xenobiotics [7]. ABC transporters are crucial in the development of multidrug resistance, with the ATP binding sites exploitable as targets for chemotherapeutic agents [8]. The mechanism of action in multidrug transportation is unclear. However, one model called hydrophobic vacuum cleaner states that, in P-glycoprotein, the drugs are bound indiscriminately from the lipid phase based on their hydrophobicity [9].

The second evolutionary diverse functional class of ATP binding proteins is called universal stress proteins (USPs). The universal stress proteins (USPs) are found in diverse group of organisms like archaea, eubacteria, yeast, fungi, and plants; their expressions are triggered by variety of environmental stressors [10]. These stressors might include but are not limited to starvation of nutrients such as carbon, nitrogen, phosphate, sulfate and the required amino acid and variety of toxicants and other agents such as heavy metals, oxidants, acids, heat shock, DNA damage, phosphate, uncouplers of the electron transport chain, and ethanol [11, 12]. The USPs bind to ATP through the ATP binding motif [G-2X-G-9X-G(S/T)] [13]. Members of the USPs will segregate into two groups based on whether or not they bind to ATP [13].

Experimental efforts are underway to determine the function of newly discovered proteins [14], but these experimental methods are costly and time consuming and at times are unsuccessful, due to the complexity involved in protein crystallization process. Several methods had been studied based on predicting ATP binding residues from their known structural features but with low accuracies [15, 16]. Some predictors of ATP binding proteins have been developed with promising results such as those in [17, 18], including Green et al. [19] article on an effective method to recognize ATP binding proteins by testing parallel cascade identification and KNN. Unfortunately these methods were adapted to ATP binding proteins containing only the classical Walker A motif [G-4X-GK (T/S)] in their sequences. The objective of this research reported here was to introduce a classifier built from a pool of protein sequences containing both ATP binding motifs of G-4X-GK (T/S) and G-2X-G-9X-G(S/T). To achieve the objective, support vector machine (SVM) approach is proposed which predicts protein functions based on the discriminative features that map protein sequences to biological functions [20–23] using the sequence pool ATP hybrid motifs.

There is a need to develop an automated predictor for ATP binding USP encoded proteins to speed experimental designs and study how these proteins function under diverse environmental stressors. This research has developed hybrid ATP binding protein predictor using the open source LIBSVM toolbox classification. The best model was the combination of amino acid and dipeptide composition of the sequences with an accuracy of 84.57% and Mathews correlation coefficient (MCC) value of 0.693%. This model shows a striking overall performance in sensitivity (82.46%), specificity (87.00%), and precision (87.85%) with area under the ROC curve (AUC) value of 0.849219. The general trend shows that combinations of descriptors perform better and improved the overall performances of individual descriptors, particularly when combined with amino acid composition. This model provides a high probability of success for molecular biologists in predicting and selecting diverse motif groups of ATP binding proteins.

## 2. Materials and Method

### 2.1. Datasets

Balanced datasets of ATP and non-ATP binding proteins were constructed from the UniProt protein database (UniProt release 2011_11) (http://www.uniprot.org/), Protein Data Bank (http://www.rcsb.org/pdb/home/home.do), IMG/M database (http://img.jgi.doe.gov/cgi-bin/m/main.cgi), and published literatures [24–26] which contain diverse universal stress proteins.

#### 2.1.1. Extraction of Walker A Motif Dataset

A total of 2000 protein sequences which belong to Walker A motif positive dataset were retrieved. Redundancy due to homologous sequences was removed using CD-HIT [27] and PISCES [28] servers at a threshold of 25%. This threshold statistically retains adequate number of protein sequences for analysis as well as avoids bias that might result from high homology. Dataset obtained was manually reviewed through literature search and information from the protein data bank [2] to ensure they represent ATP binding proteins. A total of 100 sequences were randomly selected from the original dataset and retained for training and testing to represent Walker A motif positive (ATP binding) dataset. The Walker A motif negative dataset (non-ATP binding) was taken from Yu et al. 2006 [29]. This was the “negative” dataset used for nucleic acid binding proteins. This is because ATP binding proteins are members of nucleotide binding protein family; hence the negative dataset used in [29] for predicting nucleotide binding protein family was considered useful. Redundancy was also maintained at 25% threshold and each protein was verified to be non-ATP binding using both the literature and protein data bank information. A total of 100 sequences were also randomly selected from [29] and retained for training and testing to represent Walker A motif negative (non-ATP binding) dataset.

#### 2.1.2. Extraction of USP Protein Dataset

The extracted USP sequences were tested for the presence or absence of the G-2X-G-9X-G(S/T) motif in their sequences using the NCBI conserved domain search tool [30]. The USP sequences were divided into two groups based on the presence or absence of ATP binding motif [13].The redundancy was also maintained at 25% threshold and 100 sequences were selected for each class of proteins (200 sequences in total).

The overall summary of the data prepared for analysis was as follows: (i) 100 ATP binding proteins with Walker A motif; (ii) 100 without ATP binding proteins without Walker A motif, (iii) 100 USP sequences with ATP binding motif [G-2X-G-9X-G(S/T)], and (iv) 100 USP sequences without ATP binding motif [G-2X-G-9X-G(S/T)].The 400 sequences were separated into two hybrid groups as follows: 200 ATP binding sequences and 200 sequences without ATP binding motifs and were used to generate the feature vector. The feature vector was generated from the entire sequences of the proteins (not only the ATP-binding domains) via PROFEAT server using 1497 descriptor set [31]. Physicochemical and sequence attributes of biologically informative were prioritized for investigation. The attributes were incorporated into LIBSVM classifier to find the best hybrid model for predicting ATP binding proteins.

### 2.2. LIBSVM Classifier

Support vector machines (SVM) recognized objects to be classified as points in a high-dimensional space needing a hyperplane to separate them [32].The biological molecules are represented with descriptor set. With a proper mapping furnished by a kernel function, SVM classifiers separate transformed data with a hyperplane in a high-dimensional space to predict the correct classification of protein functional classes. SVMs have been widely used in supervised classification problems in bioinformatics, such as [33–36]. The LIBSVM package which is freely downloadable at (http://www.csie.ntu.edu.tw/~cjlin/libsvm) was adopted and used to evaluate the attributes and build the final classifier, using the radial basis function (RBF) as the kernel function [37–39].

A “grid-search” was employed to select the proper values of the parameter of RBF and the penalty parameter (*C*) of the soft margin SVM. *C* was set to 2^−5^, 2^−3^,…, 2^15^ and *γ* to 2^−15^, 2^−13^,…, 2^3^. All the combinations of *C* and *γ* were tested and the pair with the best cross-validation accuracy for each feature set or combination of feature sets was selected. A smaller *γ* value makes the decision boundary smoother. The SVM training parameter *C* is the regularization factor, which controls the tradeoff between low training error and large margin [37, 40]. Throughout this work, the parameter *C* was maintained at *C* = 4 after trial and error assessment as the best value. The optimal value of *γ* was obtained for each descriptor set for best results. The entire sets of attributes were evaluated in terms of their association with ATP binding protein and a final subset with good predictive power was selected. In this research a 10-fold cross validation (10CV) was implemented. The objective of training is to maximize the ability of the SVM predictor to discriminate between classes while avoiding overfitting.

### 2.3. Tenfold Cross-Validation Analysis

The technique to evaluate any newly developed method has become a major challenge to investigators. The jack-knifing leave-one-out cross-validation (LOOCV) [41–43] is the popular technique for evaluating models. During this procedure one sequence is used for testing and the left over sequences are used for training. This process is repeated many times and each sequence is used once for testing. Even though this method is popular, it is computer intensive with considerable labor time.

In this work, 10-fold cross-validation was used to train and test the dataset with sequences randomly partitioned into ten sets. This cross-validation ensures that the dataset was split at the protein level in addition to the stratified partition, thus ensuring a more rigorous evaluation. During the procedure, the positive and negative data samples are distributed randomly into 10 sets or the so-called fold. In each of the 10 round steps, 9 of the 10 sets are used to construct a classifier (training), and then the classifier is evaluated using the remaining set (testing). This procedure was repeated ten times in a manner where each set was used for testing [44, 45]. The overall performance was the average of the performances of all the 10 sets.

### 2.4. The LIBSVM Performance Evaluation

The standard parameters used in evaluating the performance of the LIBSVM are indicated below. The overall accuracy (Acc) is the intuitive measurement of the performance on a balance dataset where as Matthew’s correlation coefficient (MCC) [46] is more realistic than Acc in measuring performance when using an unbalanced dataset [47, 48]. When both MCC and Acc values are high, the overall performance of the predicted model is better. In addition to Acc and MCC, the following parameters below were also calculated. Sensitivity is the percentage of correctly predicted binding proteins to the total binding proteins.

True positive (TP).
True negative (TN).
False positive (FP) (false alarm).
False negative (FN).
False positive rate (FPR).
Sensitivity/recall or True positive rate (TPR) TPR = TP/P = TP/(TP + FN).
Precision = TP/(TP + FP).
Accuracy (Acc) = (TP + TN)/(P + N) = (TP + TN)/(TP + TN + FP + FN).
Specificity (SPC) SPC = TN/N= TN/(FP + TN) = 1 − FPR.
Matthew’s correlation coefficient (MCC).
((TP × TN) − (FP × FN))/[sqrt ((TN + FN) × (TN + FP) × (TP + FN) × (TP + FP))] OR
  (1) $$\text{MCC}=\frac{\left(\text{TP}*\text{TN}-\text{FP}*\text{FN}\right)}{\sqrt{\text{PN}P^{\prime }N^{\prime }}}.$$

Here TP is the number of true positives (ATP-BPs), TN is the number of true negatives (non ATP-BPs), FP is the number of false positives, and FN is the number of false negatives. *2.5. Area under the ROC Curve (AUC) for LIBSVM.* It is a plot between true positive proportion (TP/TP + FN) and false positive proportion (FP/FP + TN). The StatsDirect was used package to plot ROC and calculates the area under the ROC curve directly by an extended trapezoidal rule [49]. The confidence interval was constructed using DeLong’s variance estimate [50] embedded in the statistic package.

## 3. Results and Discussion

The ATP binding proteins are known to play key roles in the biochemical functioning of the cell. In signaling pathways ATP molecules are substrates for protein kinase phosphorylation. It is difficult to identify ATP binding proteins due to lack of experimentally determined protein structures [51–53]. This is because the growth of protein sequences from various genomic projects exceeds the capacity of experimental techniques in determining protein structures and their binding reactions which are time consuming and at times unsuccessful. Therefore there is an urgent need to develop automated expert methods for determining the functional class of proteins such ATP binding proteins from their primary sequence information.

The general assumption here is that every protein that binds to ATP molecule either USPs or those having Walker A motif will have some common features embedded in their sequences. In both the USP (G-2X-G-9X-G(S/T)) and Walker A (G-4X-GK (T/S))motifs, the G, K, T, and S denote glycine, lysine, threonine, and serine, respectively, and X denotes any amino acid residue. The lysine (K) residue in the Walker A motif is crucial for nucleotide binding [54] in this class of proteins. It interacts with the phosphate groups of the nucleotide and with the magnesium ion, which coordinates the *β* - and *γ* -phosphates of the ATP molecule [55, 56].

The universal stress proteins bind to ATP through the ATP binding motif G-2X-G-9X-G(S/T), with the-G(S)/T as essential residues for ATP binding and phosphorylation [13]. Therefore, members of this class of proteins will segregate into two groups, based on whether or not they bind to ATP [13, 57]. Thus, it is important to identify ATP binding USPs and other ATP binding proteins. Several methods have been studied based on predicting ATP interacting residues if the protein structures are known, with some results showing very low accuracies [15, 16, 58, 59]. This work has predicted ATP binding proteins in general with high accuracy irrespective of their structural information using SVM classifier. The training and prediction statistics for each of the descriptor sets used were visualized and discussed below. The visualizations were constructed using Tableau Public Software (http://www.tableausoftware.com/public).

The objective in this report was to find the best descriptor set which can be use to build a predictive model for a reliable and effective server for predicting ATP-BPs in general, irrespective of their subfunctional classes. Throughout this work, the parameter *C* was maintained at *C* = 4, while the optimal value of *γ* for each descriptor was obtained and used in evaluating their performances. Their performances were evaluated based on five computed parameters consisting of their accuracies, sensitivities, specificities, precisions, and MCC, after a 10-fold cross validation (CV10).

**Figure 1:**
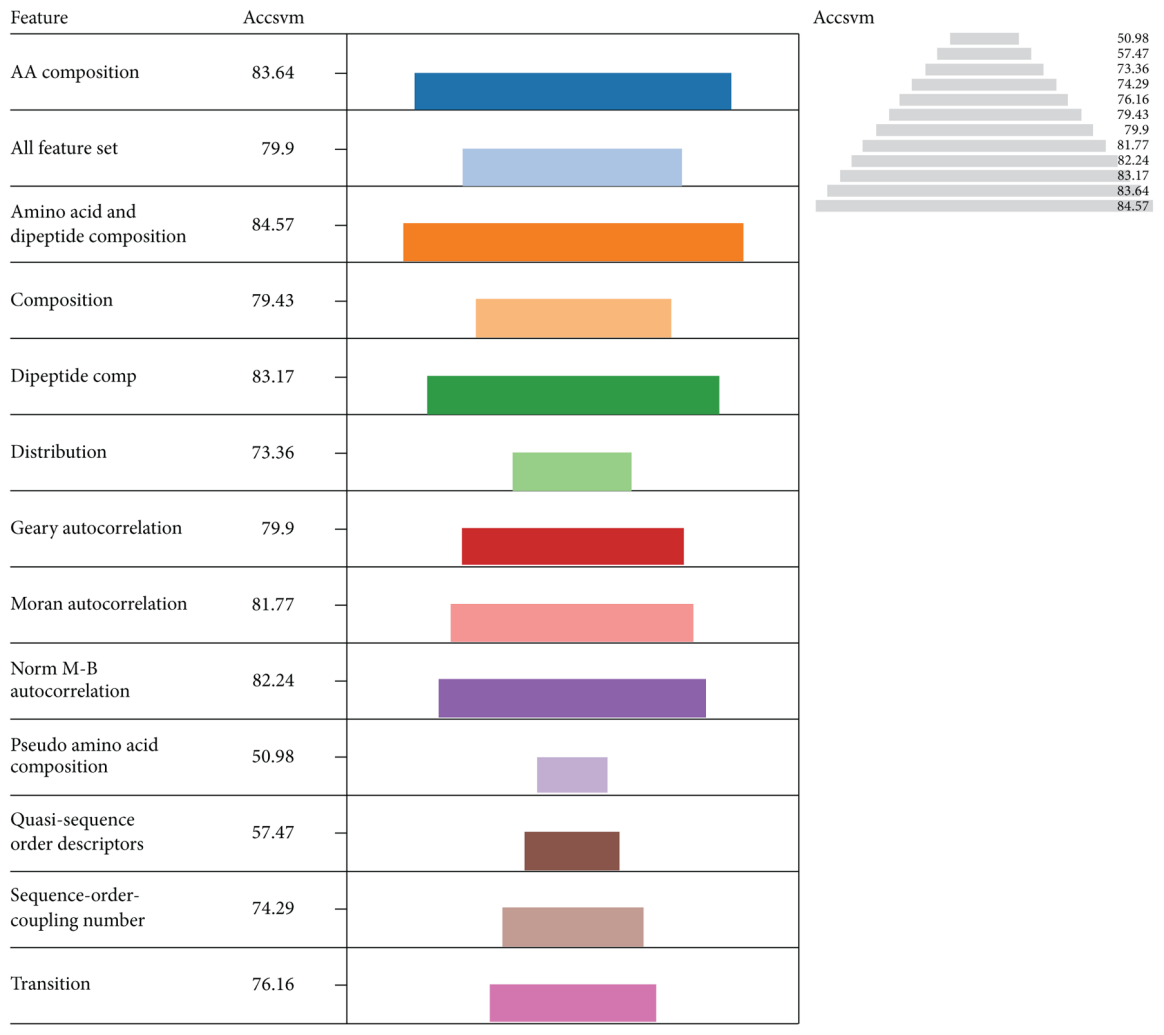
The performances of descriptors with LIBSVM in terms of accuracy The length of each color coded descriptor and the pyramidal view is a measure of their performances in terms of accuracy (Accsvm). In terms of accuracy the best descriptor was combination of amino acid and dipeptide composition (84.57%), followed by amino acid composition (83.64%), dipeptide composition (83.17%) and Norm M-B autocorrelation in that order. The pseudo amino acids and Quasi sequence order descriptors perform poorly

The performance of pseudo amino acid composition was evaluated with only accuracy due to lack of sufficient sequence information. The lengths of the color coded descriptors were used as a measure of their performances. In terms of accuracy the best descriptor was the combination of amino acid with dipeptide composition (84.57%), followed by amino acid composition alone (83.64%), dipeptide composition (83.17%), and Norm M-B autocorrelation in that order (Figure 1).The pseudo amino acids and Quasi sequence order descriptors performed poorly compared to the other descriptors. However, the overall performances of the other descriptors were better as most of them registered accuracy values greater than 70.00%. These high performers might be due to the rigorous refinement of protein sequences. Thus protein function classification with SVM classifiers can be improved drastically using rigorously refined protein sequences.

The individual performances of amino acid composition (83.64%) and dipeptide composition (83.17%) were increased to 84.57% when both descriptors were combined together. This indicates that the combination of descriptors can enhance the individual performance of other descriptors, particularly those combining with amino acid composition. This is a binary classification problem involving a balance dataset and accuracy (Acc) is the best parameter for evaluating performance based on balance dataset where as Matthew’s correlation coefficient (MCC) is more realistic than Acc when using an unbalanced dataset [47, 48]. But when both MCC and Acc values are high, the overall performance of the predicted model is better.

**Figure 2:**
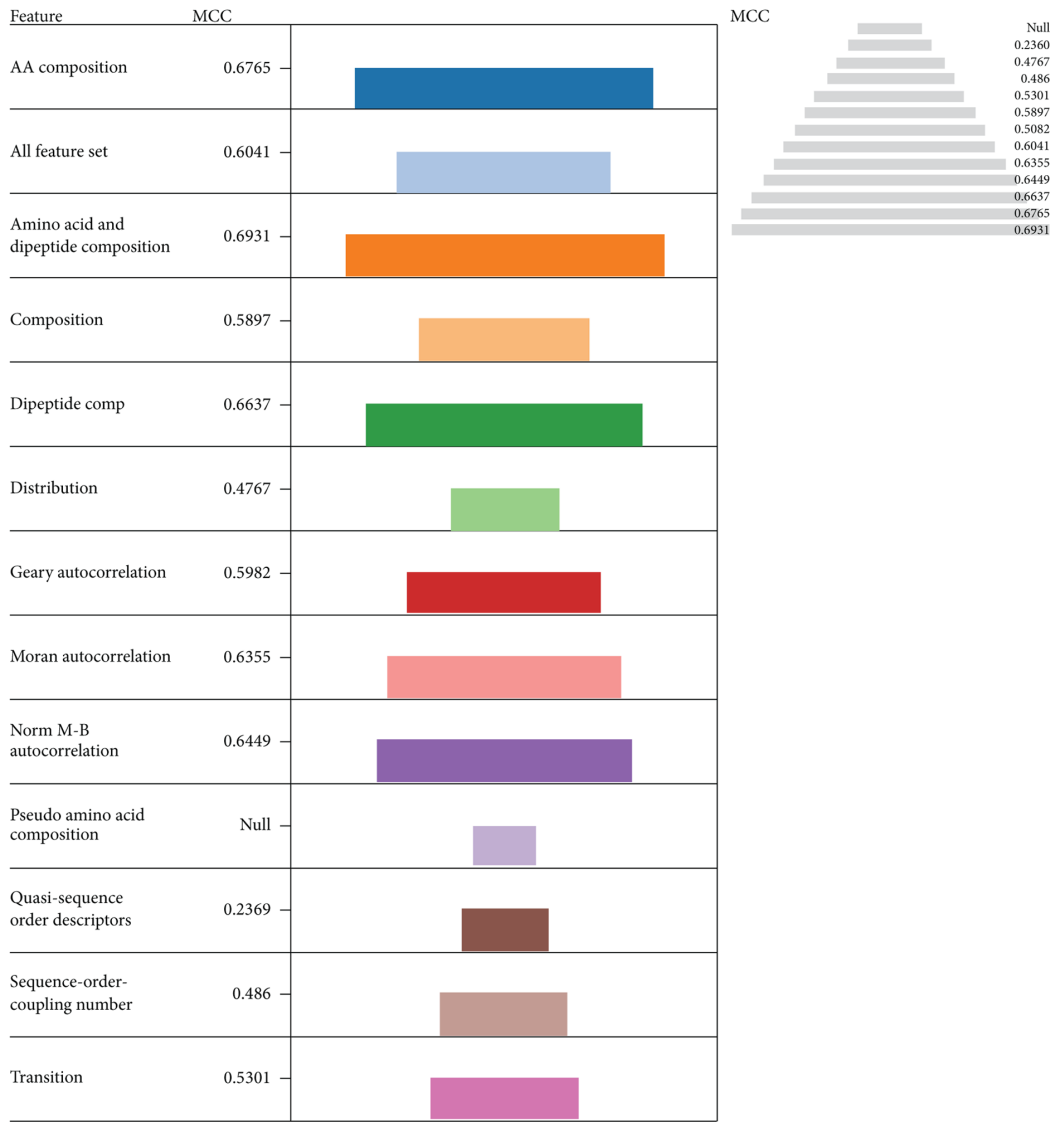
The performances of descriptors with LIBSVM in terms of Mathew’s Correlation Coefficient (MCC) The length of each color coded descriptor and the pyramidal view is a measure of their performances in terms of MCC. The best performer was amino acid and dipeptide composition in combination (0.6931) followed by amino acid composition (0.6765), dipeptide composition (0.6637) and Norm M-B autocorrelation (0.6449) in that order.

The performances of the models were evaluated based on MCC (Figure 2). The pyramidal view and the length of the color coded descriptors were used for performance visualization. The best performer was amino acid and dipeptide composition in combination (0.6931) followed by amino acid composition (0.6765), dipeptide composition (0.6637), and Norm M-B autocorrelation (0.6449) in that order. This order is in line with their performances measured using accuracy as the parameter. This result justifies the performance of the overall model. In general the combination of descriptor sets performs better than individual descriptors, particularly when combined with amino acid composition.

Therefore from the statistical point of view the use of combination sets particularly with amino acid composition tend to give better prediction performance than individual-sets [53]. The amino acid composition generally increases the overall accuracies of other descriptors in combination. One of the shortcoming of amino acid composition as a descriptor is that the same amino acid composition may correspond to diverse sequences due to the loss of sequence order [28, 60]. This sequence order information can be partially covered by combination with dipeptide composition, but dipeptide composition itself lacks information on the fraction of the individual residue in the sequence, as such a combination set is expected to give a better prediction result [27, 61] as shown above due to masking effect.

**Figure 3:**
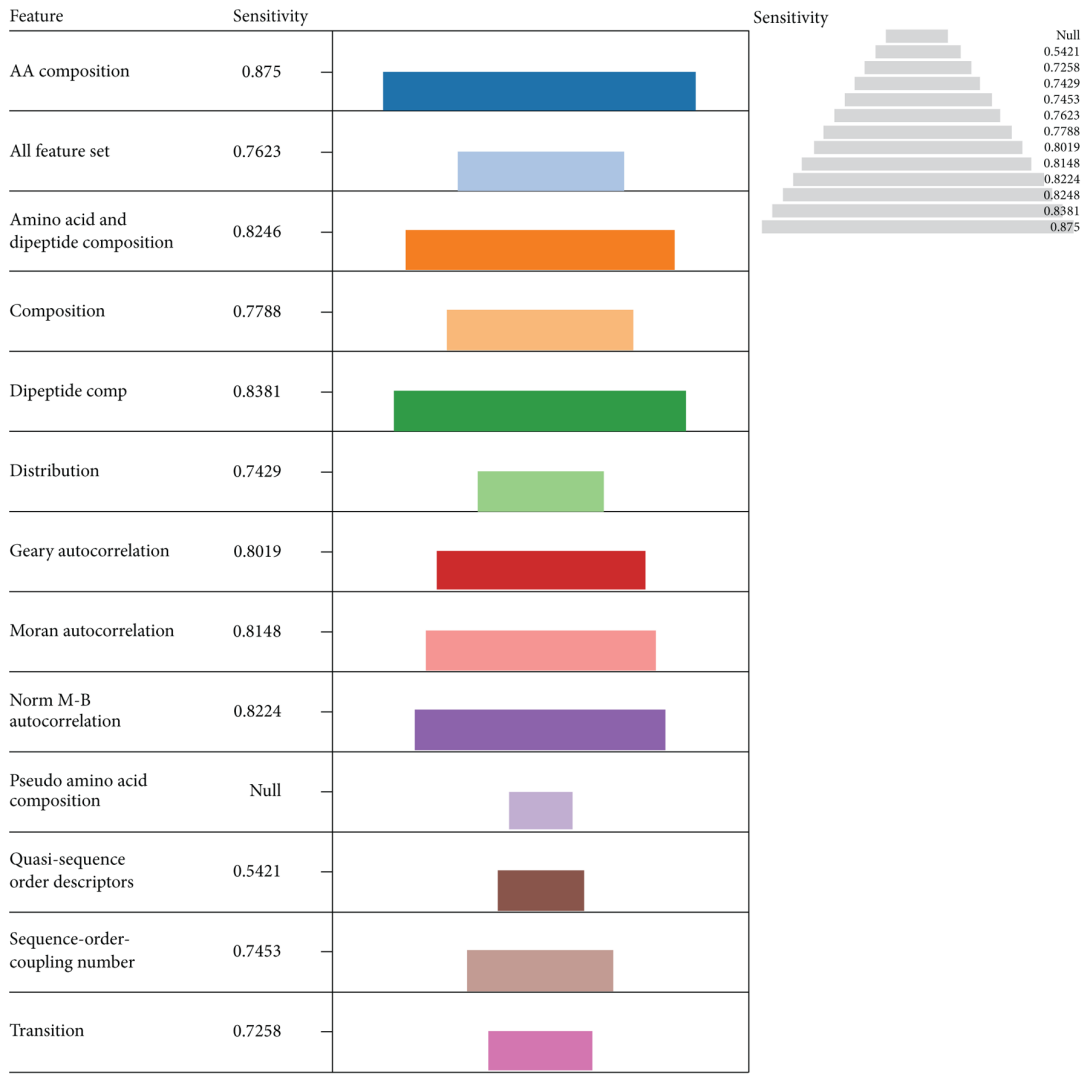
The performances of descriptors with LIBSVM in terms of Sensitivity The length of each color coded descriptor and the pyramidal view is a measure of their performances in terms of sensitivity. The most sensitive descriptor was amino acid composition (0.875) followed by dipeptide composition (0.8381), amino acid and dipeptide composition in combination (0.8246) and Norm M-B autocorrelation (0.8224) in that order.

The models were further investigated based on their sensitivity to predict ATP-BPs and the results displayed in pyramidal view (Figure 3). The most sensitive descriptor was amino acid composition (0.875) followed by dipeptide composition (0.8381), amino acid/dipeptide composition in combination (0.8246), and Norm M-B autocorrelation (0.8224) in that order.

**Figure 4:**
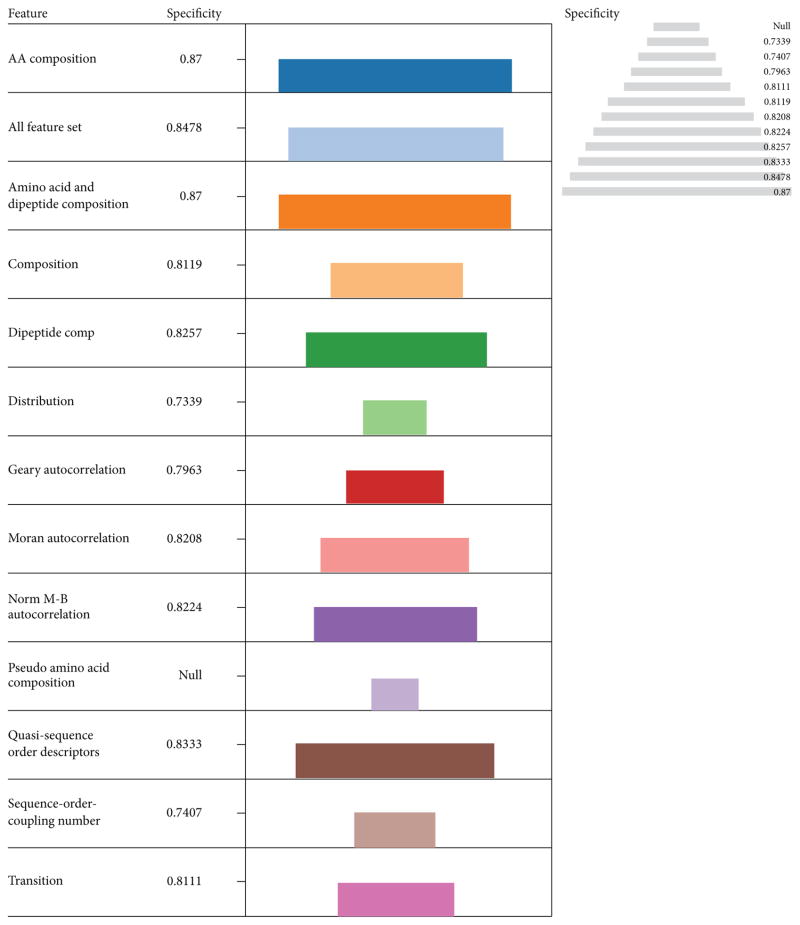
The performances of descriptors with LIBSVM in terms of Specificity The length of each color coded descriptor and the pyramidal view is a measure of their performances in terms of specificity. The most specific descriptor was amino acid composition and amino acid/dipeptide composition (0.87) followed by all using all the feature set (0.8478), Quasi sequence order descriptors (0.8333) and dipeptide composition (0.8257) in that order.

**Figure 5:**
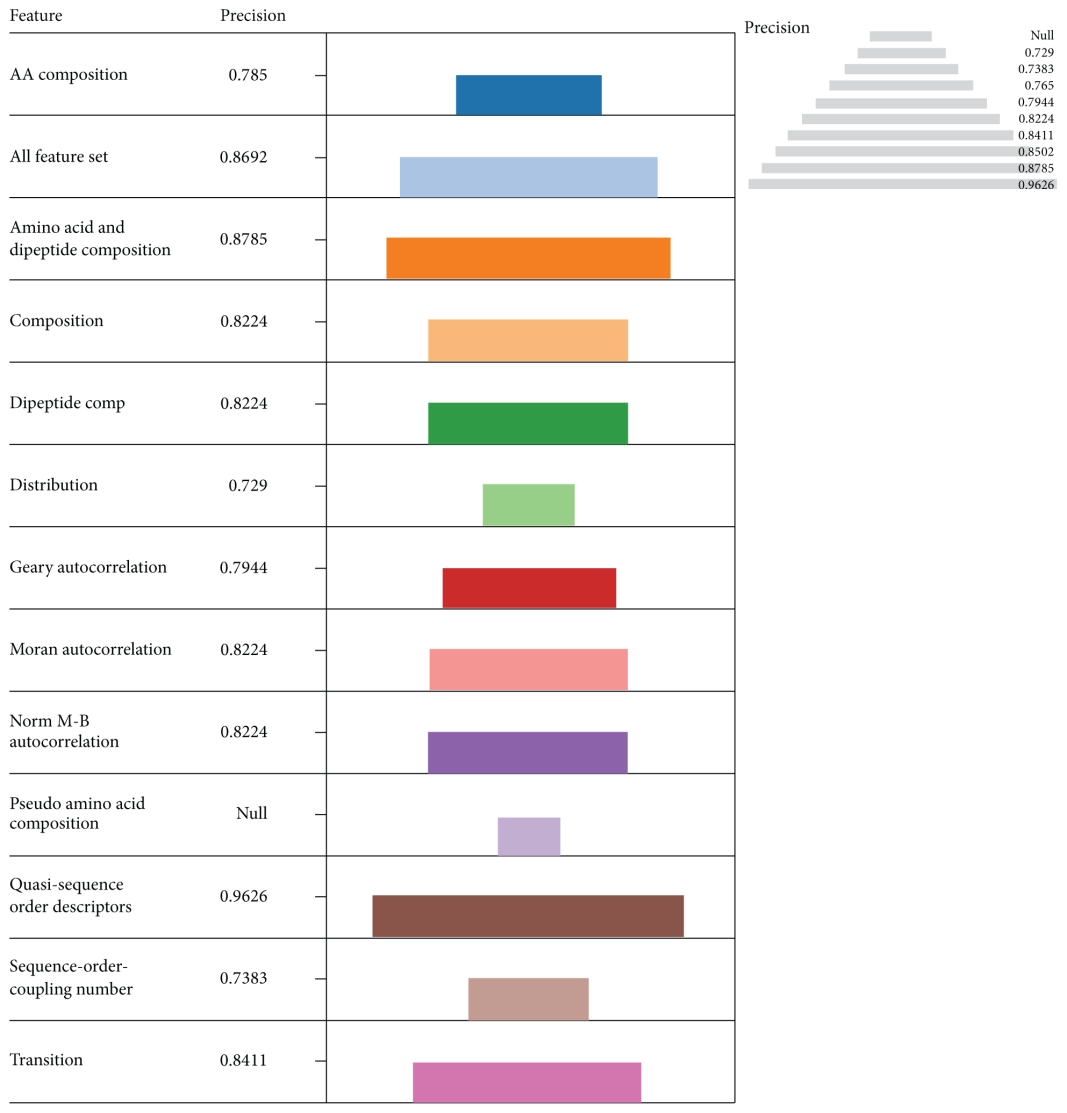
The performances of descriptors with LIBSVM in terms of Precision The length of each color coded descriptor and the pyramidal view is a measure of their performances in terms of precision. The most precise descriptor was Quasi sequence order descriptors (0.9626) followed by amino acid and dipeptide composition in combination (0.8785), all feature set (0.8692) and Transition (0.8411) in that order.

These descriptors were among the best four performers in terms of Acc and MCC. Evaluation based on specificity indicates that amino acid composition (0.87) was more specific followed by using the entire feature set (0.8478), Quasi sequence order descriptors (0.8333), and dipeptide composition (0.8257) in that order (Figure 4). This information highlights the vital role played by amino acid composition in protein function predictions in general. Interestingly the Quasi sequence order descriptors (0.9626) had the highest precision followed by amino acid and dipeptide composition in combination (0.8785), entire feature set (0.8692), and Transition (0.8411) in that order (Figure 5).

**Figure 6:**
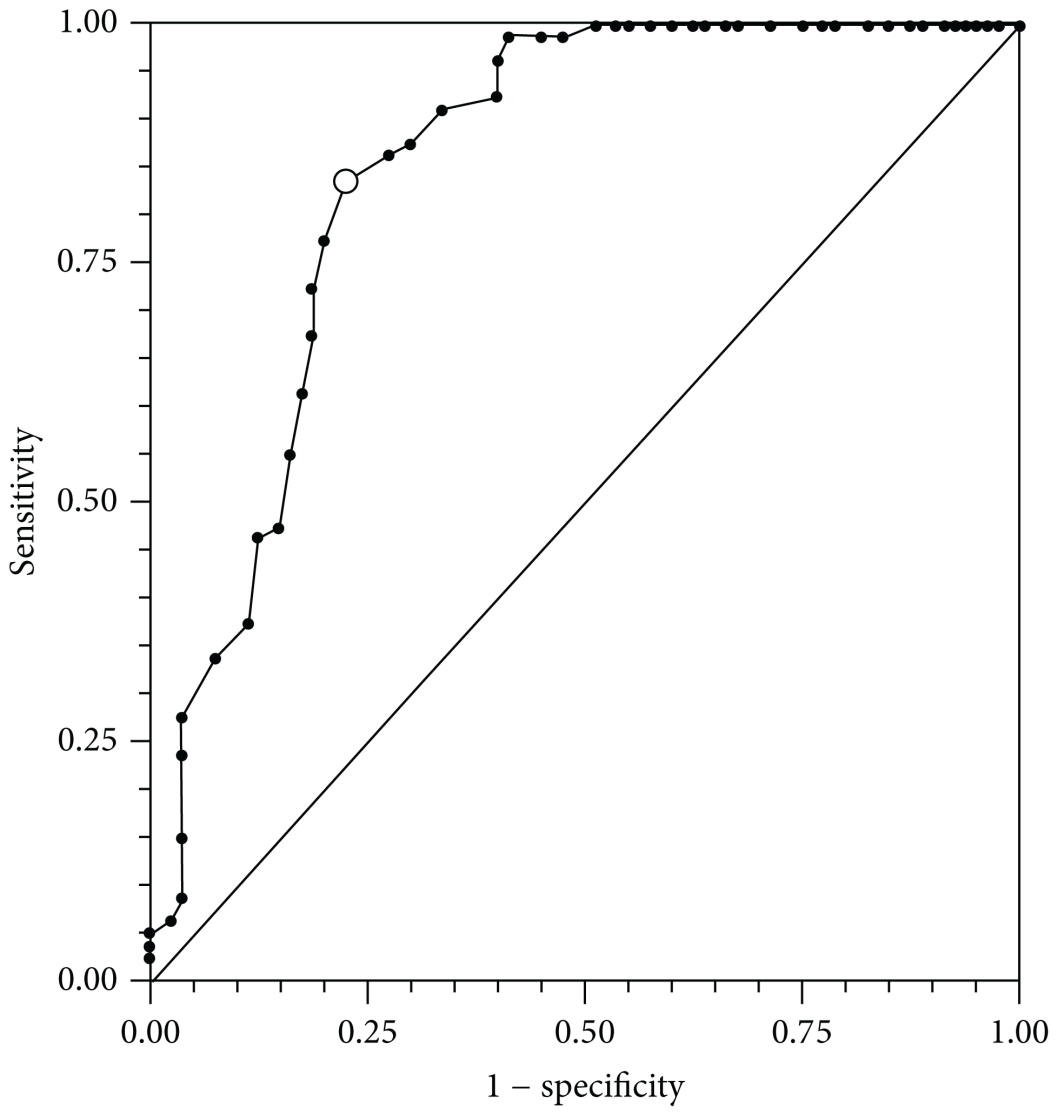
The ROC plot The plot shows the performance of the LIBSVM model generated with StatsDirect package using an extended trapezoidal rule and a non-parametric method analogous to the Wilcoxon/Mann-Whitney test to calculate the area under the ROC curve. The calculated AUA was 0.849219.

The overall model evaluation shows that the amino acids and dipeptide composition was the best model for predicting ATP-BPs from diverse functional classes using whole sequence information. The use of “all the descriptor” set did not generally result in a better model in classification. The “all features” descriptor accuracy was 79.9% against 84.57% for amino acids/dipeptide in combination. This finding is in accordance with [62, 63], on their work on molecular descriptors for predicting compounds of specific properties using “all features” set. The reduction in accuracy might be due to noise generated by the use of many overlapping and redundant descriptors. Hence the accuracy of the classifier algorithms can be severely degraded by the presence of noisy or irrelevant features, or if the feature scales are not consistent with their importance in solving the classification problem in question. The performance of the SVM model using ROC plot (Figure 6) has a value of AUC of 0.849219. This highlights a better model based on whole sequence analysis.

## 4. Conclusions

The prediction of ATP-binding proteins has been exploited using a battery of descriptor sets and a hybrid functional group. Also for the first time the prediction of ATP binding in universal stress proteins had been investigated using the support vector machine. The best hybrid model was the combination of amino acid and dipeptide composition of the sequences with an accuracy of 84.57% and Mathews correlation coefficient (MCC) value of 0.693. The general trend is that combination of descriptors will perform better and improve the overall performances of individual descriptors, particularly when combined with amino acid composition. This model provides a high probability of success for molecular biologists in predicting and selecting diverse groups of ATP binding proteins.
